# Comparative cross-methodological analysis of the IDH-wildtype glioblastoma tumor microenvironment

**DOI:** 10.1007/s00432-026-06428-6

**Published:** 2026-02-28

**Authors:** Pinar Cakmak, Jennifer H. Lun, Miriam Köhler, Michael C. Burger, Jadranka Macas, Tatjana Starzetz, Marcel H. Schulz, Yvonne Reiss, Karl H. Plate, Katharina J. Weber

**Affiliations:** 1 Goethe University Frankfurt, University Hospital, Institute of Neurology (Edinger Institute), Frankfurt, Germany; 2 Frankfurt Cancer Institute (FCI), Frankfurt, Germany; 3 Goethe University Frankfurt, University Hospital, University Cancer Center (UCT), Frankfurt, Germany; 4 Goethe University Frankfurt, University Hospital, Dr. Senckenberg Institute of Neurooncology, Frankfurt, Germany; 5 Goethe University Frankfurt, Institute for Computational Genomic Medicine, Frankfurt, Germany; 6 German Cancer Consortium (DKTK), Partner Site Frankfurt/Mainz, German Cancer Research Center (DKFZ), Heidelberg, Germany

**Keywords:** Glioblastoma, IDH-wildtype, Recurrent glioblastoma, Immune cell profiling, Tumor microenvironment, Multimodal analysis, Method comparison, Tumor deconvolution

## Abstract

**Purpose:**

Glioblastoma, IDH-wildtype is a highly aggressive and often recurrent brain malignancy characterized by a profoundly immunosuppressive and heterogeneous tumor microenvironment. In this study, we aimed to systematically compare commonly used immune profiling methodologies.

**Methods:**

We conducted a cross-platform comparison using matched primary and recurrent tumor samples analyzed by immunohistochemistry, multiplex immunofluorescence, AI-driven image analysis, DNA methylation profiling, and bulk RNA sequencing. A total of 72 samples from 36 patients were evaluated to assess cross-method concordance, cell-type resolution, and each platform’s ability to capture TME dynamics throughout disease progression.

**Results:**

Across modalities, monocyte/macrophage-lineage cells were the most consistently identified and quantifiable population. Image-based techniques, including immunohistochemistry, multiplex immunofluorescence, and AI-driven quantification, demonstrated strong concordance for B cell and macrophage detection, whereas T cell quantification showed greater inter-method variability, particularly in recurrent tumors. RNA sequencing-based deconvolution captured broader spectrum of immune and neoplastic states, including microglial enrichment, but aligned only moderately with protein-level measurements. DNA methylation-based approaches performed robustly for myeloid cell estimation but limited accuracy for lymphocyte populations.

**Conclusion:**

This study highlights the complementary strengths and limitations of current immune profiling modalities in GB. An integrative, method-aware framework facilitates more accurate immune cell quantification and deeper biological insights into TME evolution, ultimately informing the development of precision immunotherapeutic strategies for recurrent GB.

**Supplementary Information:**

The online version contains supplementary material available at 10.1007/s00432-026-06428-6.

## Introduction

Glioblastoma, IDH wildtype (CNS WHO grade 4, GB) remains one of the most aggressive human cancers. Its profoundly immunosuppressive and heterogeneous tumor microenvironment (TME) promotes immune evasion, therapeutic resistance, and rapid disease progression (Faisal et al. 2024; Read et al. 2024; Zhang et al. 2023). The GB TME is dominated by myeloid populations—particularly M2-polarized glioma-associated macrophages and microglia—and is notably deficient in lymphocytes, contributing to the establishment of a highly suppressive immune milieu. This landscape is further shaped by marked spatial and temporal heterogeneity across and within tumors (Read et al. 2024; Zhang et al. 2023; Ho et al. 2024). Such immune-suppressive behavior arises not only from intrinsic tumor cell signalling, but also from disease-specific recruitment and activation of immune cell populations.

Multiple strategies have emerged for characterizing immune features within the GB TME. While no single approach fully captures its breadth and complexity, histology-based techniques remain central for spatial analysis of immune architecture, particularly for characterizing glioma-associated microglia and macrophages in anatomically and functionally distinct tumor regions (Dadario et al. 2024). Furthermore, DNA methylation-based subclassification has revealed distinct immunological profiles among GB phenotypes, with mesenchymal subtypes showing enhanced T-cell infiltration and characteristic cytokine expression patterns (Dejaegher et al. 2021; Drexler et al. 2024; Klughammer et al. 2018). Importantly, these subclasses are dynamic: spatial and longitudinal profiling of matched primary and recurrent tumors has uncovered non-linear immune evolution, including macrophage expansion and region-specific shifts in immune marker expression (Loussouarn et al. 2023; Maddison et al. 2021). Despite these insights, the clinical impact of immunotherapeutic strategies remains limited, reflecting the complexity and resistance of the GB immune microenvironment.

Concurrently, AI-driven methodologies—including convolutional neural networks (CNNs) and advanced segmentation algorithms—are emerging as powerful tools for automated, reproducible quantification of immune infiltration, offering scalability and precision in analysing complex tissue architecture (Baharun et al. 2024). Computational tools such as ‘GBMdeconvoluteR’ enable the partitioning of bulk RNA-seq data into immune and neoplastic components by integrating neoplastic reference signatures with single-cell immune atlases. This approach effectively recapitulates known links between mesenchymal features and immune infiltration, offering refined perspectives on tumor progression and prognosis (Ajaib et al. 2023). The integration of in situ approaches, such as immunohistochemistry (IHC) and multiplex immunofluorescence (mIF), which preserve spatial resolution, with in silico strategies capable of high-dimensional molecular profiling enables a more holistic and comprehensive understanding of the GB TME (Read et al. 2024; Moon et al. 2023). This integrative perspective is essential for uncovering the spatial and functional diversity of immune–tumor interactions. Here, we present a comprehensive, integrative evaluation of immune characterization strategies in GB, emphasizing validation and comparison of analytical approaches. By comparing complementary immune profiling methodologies against mIF across matched primary and recurrent IDH-wildtype tumors, we delineate both the technical boundaries and the biologically driven heterogeneity shaping the GB immune landscape. This framework establishes a foundation for reproducible immune profiling and translational research in this highly refractory malignancy.

## Materials and methods

### Sample and data collection

Electronic patient records were screened to identify individuals with GB who received primary and recurrent surgical treatment at the Dr Senckenberg Institute of Neurooncology of Goethe University Frankfurt, University Hospital, between 2013 and 2022. Formalin-fixed paraffin-embedded (FFPE) GB tissue samples were collected from the University Cancer Center (UCT) Biobank. All patients provided written informed consent, and the study was approved by the local ethics committee (SNO-2-2023).

### Histological assessment of tumor sections

Comprehensive histopathological evaluation of hematoxylin and eosin (H&E)-stained sections was performed by a board-certified neuropathologist (K.J.W.) and a pathologist (P.C.). The proportions of viable tumor, infiltration zones, normal brain tissue, fibrosis, necrosis, hemorrhage, calcification, and reactive/resorptive areas were systematically estimated using a high-resolution light microscope (model BX51; Olympus, Japan).

### Tumor classification and DNA methylation profiling

IDH1 R132H IHC was performed for all cases to confirm IDH wildtype status. For patients under 55, DNA methylation profiling was added to exclude IDH mutant methylation classes. No IDH mutations were detected by both methods. In a subset of cases, methylation data was also used to assess copy number aberrations, including *EGFR* amplification and chromosomal +7/− 10 changes.

### Immunohistochemistry and immune cell scoring

IHC staining was performed on the entire cohort to analyze immune cell composition within the TME. Tissue Sects. (3 µm) were stained on an automated Leica stainer (SM2000R, Leica, Germany) using the following antibodies: anti-CD3 (polyclonal Rabbit Anti-human CD3, 1:500 dilution; DAKO, Denmark), anti-CD4 (clone 4B12, catalog no. PA0427; Leica), anti-CD8 (clone C8/144B, 1:100 dilution; DAKO), anti-CD20 (clone L26, 1:500 dilution; DAKO), anti-CD163 (clone 10D6, 1:500 dilution; Novus Biologicals, USA), and anti-Iba1 (polyclonal, 1:1000 dilution; Wako, Japan). Staining followed standard Leica SM2000R automated protocols.

A pathologist (P.C.) and a board-certified neuropathologist (K.J.W.) evaluated the stained slides using a bright-field microscope (model BX51; Olympus), estimating the percentage of positive cells for each marker relative to the total cells in the whole-mount tumor sections.

### mIF staining and digital image analysis

To characterize immune cell populations, mIF staining was performed on the entire cohort using the Opal Polaris 7-color manual detection kit (NEL861001KT; Akoya Biosciences, USA), which relies on the Tyramide Signal Amplification (TSA) technique. The staining was conducted on a LabSat^®^ Research automated staining device (Lunaphore Technologies SA, Switzerland), with an antibody panel targeting CD3 (#A0452; DAKO), CD8 (#M7103; DAKO), CD163 (#ab265592; Abcam), and Iba1 (#019-19741; Wako). Nuclear staining was performed using DAPI (4´6-diamidino-2-phenylindole, SKU FP1490; Akoya Biosciences).

Multiplex images were acquired at 0.5 µm/pixel using the Vectra Polaris™ Automated Quantitative Pathology Imaging System (Akoya Biosciences) with MOTiF technology. Whole-slide multispectral images were processed with ‘PhenoChart’ (Akoya Biosciences) for visualization, InForm^®^ (Akoya Biosciences) for spectral unmixing and batch analysis and HALO^®^ (Indica Labs) for image fusion and downstream analysis. Percentages of CD3^+^, CD8^+^, and CD163^+^ cells were quantified relative to the total cells in whole slide; CD20^+^ cell fractions were quantified using HALO brightfield (HALOBF) mode. Paired primary GB (pGB) and recurrent GB (rGB) samples were compared using the Wilcoxon signed-rank test.

### AI-based cell segmentation pipeline

Cell segmentation and quantification of whole-slide brightfield IHC images were performed using a fully automated AI-driven pipeline developed and validated in-house, as previously described by Köhler et al. (2024). The workflow consists of two main steps: (1) tumor region detection using a ResNet-34-based classifier on 200 × 200-pixel tiles (He et al. 2016), and (2) pixel-wise cell segmentation using a U-Net model with a ResNet34 backbone (Ronneberger et al. 2015) to identify background, hematoxylin-only nuclei, and immuno-stained immune and stromal cells. Segmented objects were quantified using connected-component analysis, and immune-cell ratios were computed per slide. For CD3, CD4 and CD8 stains, regions with > 0.75 immune-cell fraction were excluded to reduce artefactual overcalling.

Segmentation performance, assessed using the Dice similarity coefficient against manual annotations, achieved mean scores of 0.84 ± 0.05 for CD163^+^ macrophages and 0.72 ± 0.08 for CD8^+^ T cells. Comparisons with ‘Cellpose’ (Stringer et al. 2021) and a classical Watershed algorithm (Roerdink and Meijster 2000) showed strong performance for abundant and morphologically distinct cell types (e.g., macrophages), and acceptable accuracy for sparse T-cell populations.

### DNA methylation analysis and data analysis

For DNA methylation analysis, tumor regions with highest tumor cell density were selected on H&E-stained sections. DNA was extracted from 2 mm-diameter FFPE punch biopsies using either the Stratek Invisorb Genomic DNA Kit II (Stratek molecular, Germany) or the Maxwell RSC FFPE Plus DNA Kit (Promega, USA), following the manufacturer’s protocols. DNA concentration was measured using the Qubit DNA BR Assay Kit and Qubit 3 Fluorometer device (Invitrogen, Thermo Fisher Scientific, USA). DNA was processed and hybridized to the Infinium™ MethylationEPIC v1.0 Beadchip (Illumina, USA) following the manufacturer’s protocols and scanned on the iScan system (Illumina) to generate raw intensity data (IDAT) using the GenomeStudio software (Illumina).

IDAT files were uploaded to molecularpathology.org (formerly provided by the University of Heidelberg, Germany) for classifier results, MGMT promoter methylation status, and copy number analysis using the Heidelberg brain tumor classifier (versions 11b4 and v12.5). Reference-based tumor deconvolution was performed with the R package ‘MethylCIBERSORT’ (Chakravarthy et al. 2018), comparing cell type-specific methylation profiles—including tumor cells, immune cell subsets (CD4^+^ effector T cells, CD8^+^ T cells, CD14^+^ monocytes, CD19^+^ B cells, CD56^+^ NK cells, eosinophils, neutrophils and regulatory T cells), neurons, and glial cells—to tumor methylomes. IDAT files were processed in R with package ‘minfi’ for quality control, NOOB normalization, and beta values acquisition. The resulting beta value matrices were submitted to the CIBERSORT website to perform cellular deconvolution (Newman et al. 2015).

### Bulk RNA-sequencing and data analysis

A total of 38 GB samples (19 pairs of pGB and rGB) underwent bulk RNA sequencing. Tumor regions were annotated by a pathologist (P.C) on H&E- stained slides and microdissected in 10 × 10 µm sections. RNA was extracted using the Maxwell RSC FFPE RNA Kit (Promega), quantified with Qubit 3.0 Fluorometer with the Qubit RNA BR Assay Kit (Thermo Fisher Scientific, USA) and assay for quality on the TapeStation 4150 system with High Sensitivity RNA ScreenTape (Agilent Technologies, USA). Libraries were prepared with the Illumina Total RNA Prep Ligation with Ribo-Zero Plus kit (Illumina) using 200 ng RNA for ribosomal RNA (rRNA) depletion, fragmentation, complementary DNA (cDNA) synthesis, library clean-up with AMPure XP beads (Beckman Coulter, USA), and amplification. Sequencing was performed on an Illumina NextSeq 1000 Sequencer with 100 bp paired-end reads, using NextSeq 1000 P2 200-cycle flow cells.

Reads were processed using the DRAGEN RNA pipeline (v4.0.4, Illumina) against the human genome version 38 (alt-masked v3, graph enabled). Normalized counts were combined with clinical and sequencing metadata into a ‘SummarizedExperiment’ object in R (v4.3). After excluding long non-coding RNA and pseudogenes, 21,665 genes were retained. Differential expression was analyzed using the R package ‘DESeq2’ (Love et al. 2014), including tumor status, sex, and sequencing runs as variables. Genes with adjusted *p*-values (*padj*) < 0.05 and an absolute log_2_ fold change ≥ 1 were considered significant. Significant genes were analyzed for pathway enrichment using Gene Set Enrichment Analysis (GSEA) with the ‘clusterProfiler’ package (Xu et al. 2024).

Cell-type deconvolution was performed with ‘GBMdeconvoluteR’ (Ajaib et al. 2023). Transcripts per million (TPM) values from RNA sequencing were uploaded to the GBMdeconvoluteR interactive web interface (https://gbmdeconvoluter.leeds.ac.uk) using the authors’ marker gene list. Resulting cell-type proportions—including neoplastic cells (AC, MES, NPC and OPC) and immune cells (B cells, dendritic cells, macrophages, mast cells, microglia, monocytes, NK cells and T cells)—were imported into R for statistical analysis and visualization.

### Statistical analysis

Tumors were stratified into high and low groups for each immune marker (CD3, CD8, CD163) using the median proportion of positive cells. Spearman’s ranking correlation was used to assess method concordance in pGB and rGB samples. All statistical analyses were performed in RStudio (R Core Team, 2019), and figures were created using Affinity Designer (Affinity, UK), BioRender (BioRender, Canada), and RStudio (Posit, USA).

### Ground truth reference for cross-method validation

Semi-quantitative mIF assessment was used as the reference standard for immune cell quantification. Whole slide mIF images, simultaneously detecting multiple immune markers, were reviewed and scored by an experienced pathologist (P.C.), evaluating staining quality, tissue integrity, and autofluorescence. Ambiguous, artefactual or low-quality regions were manually excluded. The resulting mIF-derived immune cell ratios served as the benchmark to assess the accuracy of conventional light microscopy, AI-based segmentation and in silico-deconvolution methods.

## Results

### Study design, cohort characteristics and tissue composition of pGB and rGB

**Fig. 1:**
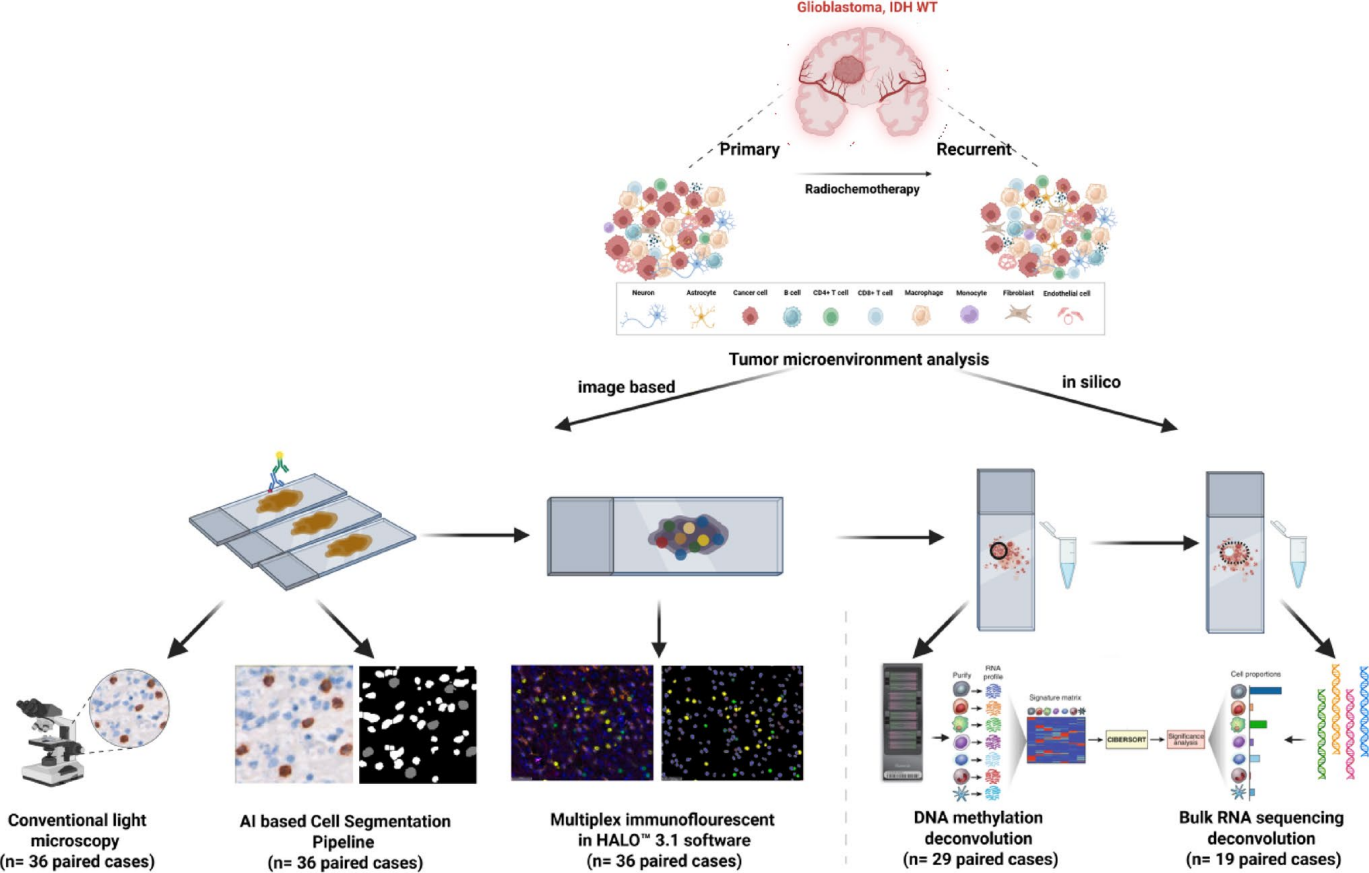
Study design and analysis of the GB TME in pGB and rGB samples. The TME composition was assessed using both image-based and in silico deconvolution approaches. This scheme illustrates tissue processing, image acquisition, and computational analysis steps used for comparative and correlative evaluation across platforms. Consecutive sections from the same FFPE tumor blocks were used for all image-based analyses. Corresponding regions were sampled for molecular profiling: tissue punches for DNA methylation and tumor-enriched regions microdissected from H&E-stained sections for RNA sequencing. Image-based analysis included conventional light microscopy, AI-based cell segmentation, and mIF. In silico-analyses involved deconvolution of DNA methylation and bulk RNA sequencing data to estimate cell-type proportions within the TME. Sample sizes (n) for each method are indicated

To investigate the method-specific outputs on estimated cellular composition of the GB TME we used image-based and in silico-deconvolution approaches in matched pairs of pGB and rGB (Fig. 1):

IHC with manual light microscopy-based estimation of cellular fractions (36 pairs), automated AI-based image analysis of IHC-stained slides (36 pairs), mIF with software-based cell quantification (36 pairs), and in silico deconvolution of bulk DNA methylation data (29 pairs) and RNA sequencing data (19 pairs). All image-based assays (IHC, AI-based segmentation, and mIF; exemplary represented in (Supplementary Fig. 1A-B) were performed on consecutive whole-slide sections from the same FFPE tumor blocks. IHC followed by estimation of cellular quantities and AI-based analyses were performed on the same set of slides. For molecular profiling, regions of high tumor cell density were sampled—tissue punches for DNA methylation analysis and consecutive microdissected areas for RNA sequencing. The established cell type and method-specific markers used for this analysis are listed in Table 1.

**Table 1:** Cell type identification methods and approaches

Cell types	Conventional microscopy	AI-Pipeline	Multiplex-IF	DNA MethylCIBERSORT	RNA GBMdeconvoluteR
T cell	CD3 CD4 CD8	CD3 CD4 CD8	CD3 CD8	CD4 eff Treg CD8	T cells
B cell	CD20	CD20	CD20	CD19	B cells
Myeloid cells	CD163 Iba-1	CD163 Iba-1	CD163	CD14	Monocytes + Macrophages

This study included a total of 72 samples, all confirmed as GB in accordance with the 2021 WHO Classification of Tumors of the Central Nervous System (Louis et al. 2021). The cohort had a median age of 55 years (range: 41–73) at initial surgery and 57.5 years (range: 42–75) at recurrence (Table 2) and comprised eight females (22.2%) and 28 males (77.8%). Tumor locations varied, with 41.7% in the temporal lobe, 11.1% in the frontal lobe, 11.1% in the parietal lobe, and 36.1% in other brain regions.

**Table 2:** Clinical information of patients within the cohort (n = 36)

Median age in years, *n (range)* First surgery Second surgery	55 (41–73) 57.5 (42–75)
Gender, *n (%)* Female Male	8 (22.2) 28 (77.7)
Tumor location, *n (%)* Temporal Frontal Parietal Other	15 (41.7) 4 (11.1) 4 (11.1) 13 (36.1)
Primary treatment, *n (%)* Rthx Rthx + TMZ Rthx + Nivo Rthx + TMZ + Nivo/Placebo Rthx + CCNU/TMZ NA	1 (2.7) 27 (75) 1 (2.7) 1 (2.7) 3 (8.3) 3 (8.3)
Median time to recurrence in month, *n (range)*	15.8 (1.5 -73.3)

For the primary treatment, majority of the patients (n = 27, 75%) received the standard of care: radiation therapy combined with temozolomide (Rthx + TMZ). The median time to recurrence was 15.8 months (range: 1.5–73.3) (Table 2).

To account for potential confounding effects arising from tissue heterogeneity, we compared the histological composition of pGB and rGB samples (Supplementary Fig. 1C, D). rGB samples contained significantly higher proportions of reactive/resorptive areas (*p* = 0.0083), fibrosis (*p* = 0.018), and normal parenchyma (*p* = 0.0058), along with a reduced fraction of necrosis (*p* = 0.0062), compared to pGBs (Supplementary Fig. 1C-D). No significant differences were observed in the fractions of vital tumor, infiltration zone, hemorrhage, or calcifications (Supplementary Fig. 1C-D).

### Monocytes exhibit the highest concordance across image-based quantification methods

**Fig. 2:**
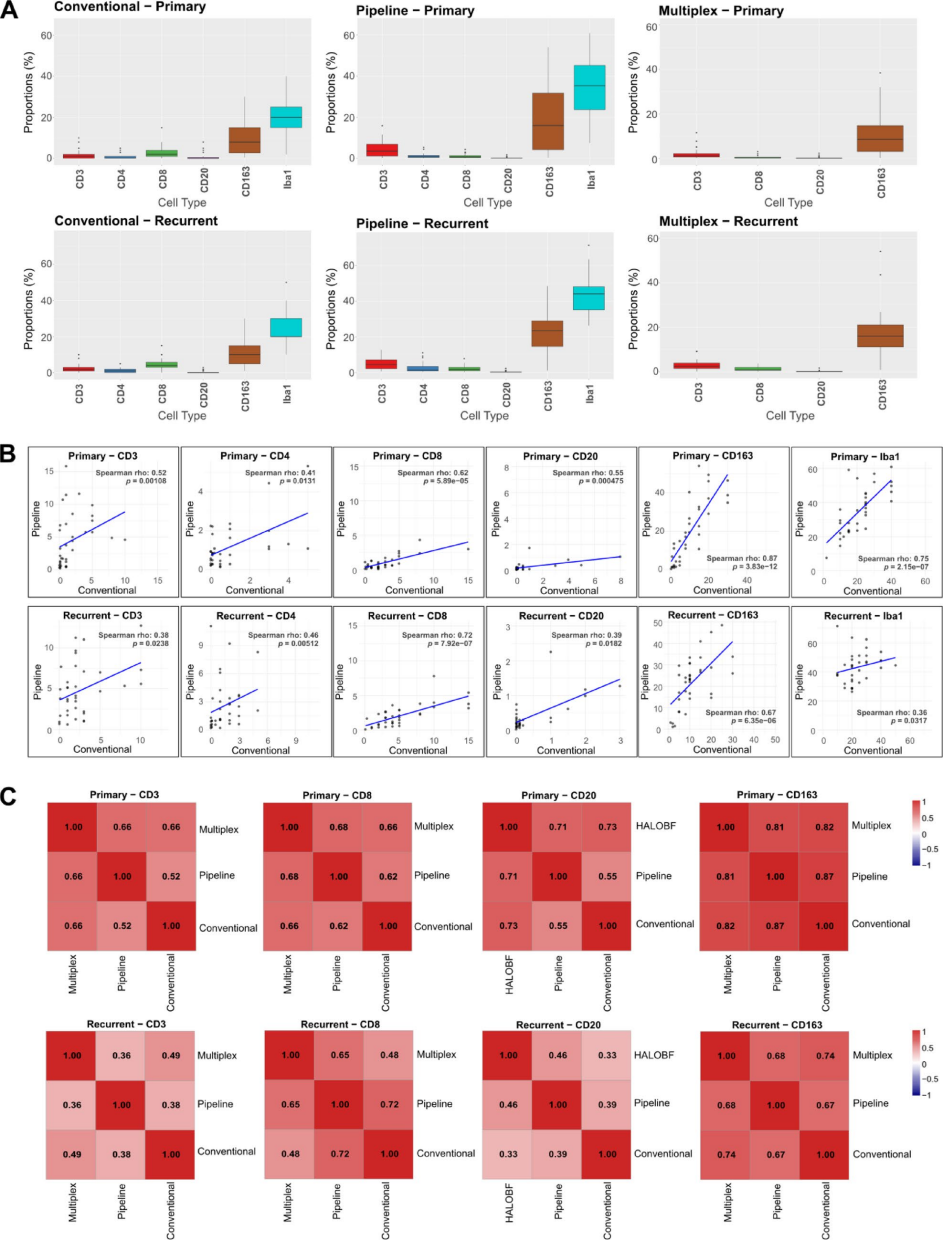
Analysis of immune cell infiltrates across image-based quantification methods and disease states. **a** Boxplots showing the quantification of immune cell populations (CD3, CD4, CD8, CD20, CD163, and Iba1) within the TME. Data is stratified by quantification methods (IHC, AI-based pipeline, and mIF) and disease state (pGB and rGB). The y-axis represents the percentage value obtained by each method. **b** Scatter plots illustrating Spearman's rank correlations between immune cell populations in pGB and rGB samples. Each plot compares conventional and pipeline quantification methods for specific markers (CD3, CD4, CD8, CD20, CD163 and Iba1). Spearman’s rho (ρ) and *p* values are shown, indicating the strength and statistical significance of each correlation. The blue line represents the line of best fit. **c** Heatmaps summarizing Spearman's rank correlation coefficients between mIF, pipeline, and IHC quantifications for CD3, CD8, CD20, and CD163 in pGB and rGB samples. Red and blue colors represent positive and negative correlation, respectively

Among all image-based quantification techniques, myeloid cells (identified by CD163 or Iba1 staining) were the most abundant immune cell type within the TME of both pGB and rGB samples (Fig. 2a). The AI-based pipeline and multiplex assays revealed an increase in myeloid populations following tumor recurrence. Quantification results from conventional light microscopy and AI-based quantification methods showed moderate to very strong, statistically significant correlations for T cell subsets (CD3, CD4, CD8), B cells (CD20), and myeloid cells (CD163, Iba1) in both pGB and rGB samples (Fig. 2b; top row for pGB, bottom row for rGB). The highest concordance between the two image-based approaches performed on the same sections was found for Iba1^+^ monocytes in pGB (ρ = 0.75), CD8^+^ T cells in rGB (ρ = 0.72), and CD163^+^ monocytes in both pGB (ρ = 0.87) and rGB (ρ = 0.67) (Fig. 2b).

In pGB samples, mIF analysis correlated strongly with both conventional and AI-based methods for lymphocyte markers (ρ = 0.66/0.66 for CD3^+^, ρ = 0.66/0.68 for CD8^+^ and ρ = 0.73/0.71 for CD20^+^; Fig. 2c, top panel) and CD163^+^ monocytes (ρ = 0.82/0.81). In rGB the concordance with mIF decreased across all cell types, with correlations ranging from ρ = 0.49/0.36 for CD3^+^ T cells to ρ = 0.74/0.68 for CD163^+^ monocytes (Fig. 2c, bottom panel).

### Discrepancies among in silico-deconvolution methods in immune cell quantification in GB

**Fig. 3:**
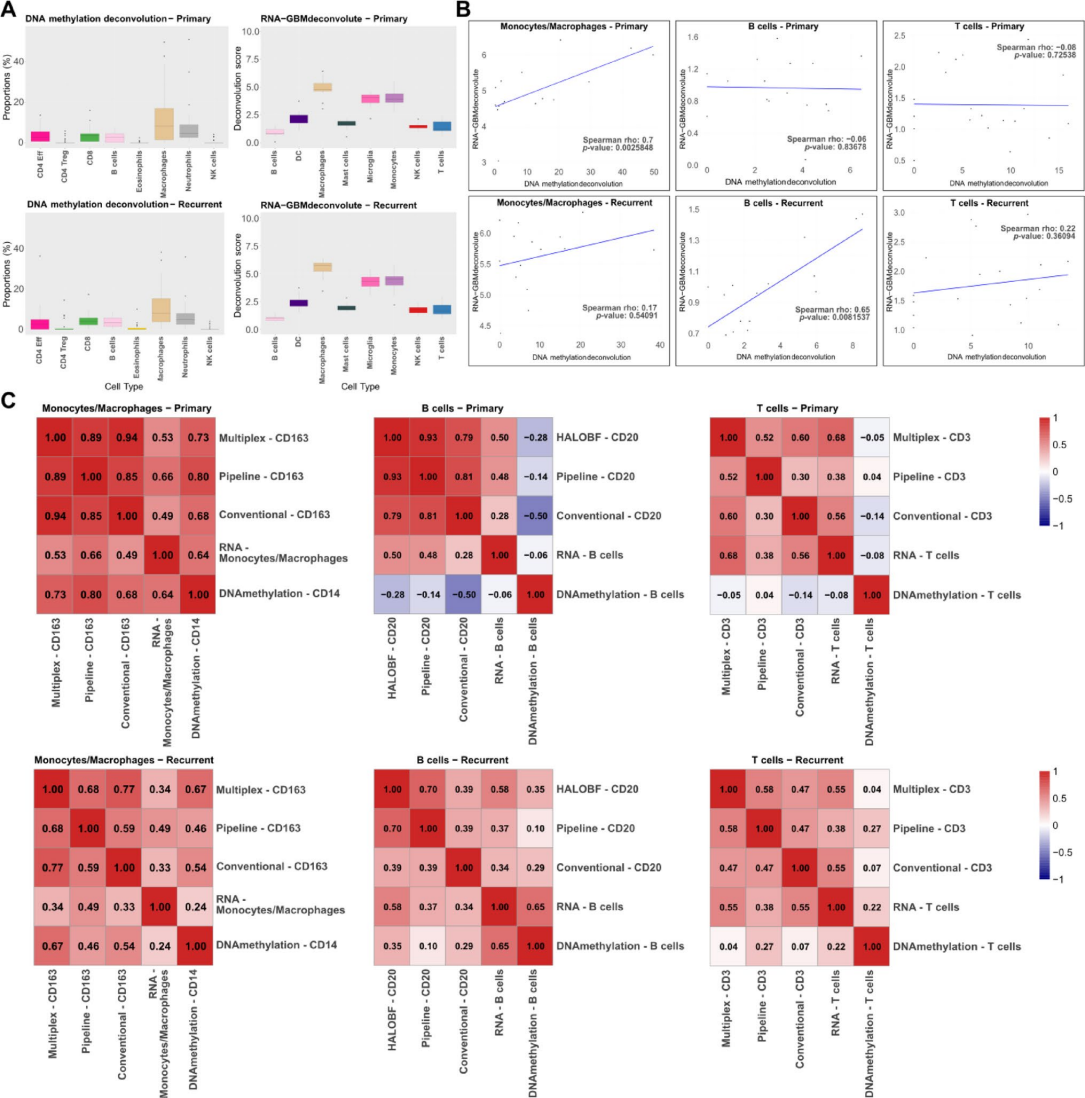
Comparison of immune cell quantification between in silico-methods and across all methods. **a** Boxplots illustrating the distribution of immune cell types identified in pGB and rGB, as estimated by DNA methylation-based deconvolution (left) and RNA-based deconvolution (right). Immune cell types are indicated on the x-axis, and the y-axis represents the estimated proportion of each cell type. **b** Scatter plots showing the Spearman’s rank correlation between DNA methylation-based deconvolution and RNA sequencing-based deconvolution for combined immune cell populations (monocytes/macrophages, T cells, and B cells) in pGB and rGB. Spearman’s rho (ρ) and *p*-values are indicated on each plot. The blue line represents the line of best fit. **c** Heatmaps displaying Spearman's rank correlation coefficients between individual quantification methods for specific immune cell type markers in pGB and rGB. The methods compared include DNA methylation deconvolution, RNA deconvolution, mIF, AI-based pipeline, and IHC approaches. Color intensity indicates the strength and direction of correlation, with red denoting positive and blue denoting negative correlations

Consistent with image-based analysis, in silico-deconvolution methods identified macrophages, monocytes and microglia as the most prevalent cell type within the TME in both pGB and rGB (Fig. 3a). Moreover, RNA sequencing-based analysis of monocyte subpopulations also revealed that microglia were among the most prevalent cell types (Fig. 3a, right panels).

In pGB samples, a strong correlation was observed for myeloid cells (ρ = 0.7, *p* < 0.05), indicating an overlap between the two methods in quantifying this population (Fig. 3b). However, no statistically significant correlations were found for myeloid cells in rGB, for B cells in pGB or for T cells overall. Of note, a strong correlation between methods was observed in B cells in rGB (ρ = 0.65, *p* < 0.01) (Fig. 3b).

### Comparative immune cell profiling reveals variable concordance across GB states

The inter-method comparison indicated robust positive correlations for monocytes/macrophages quantification in pGB samples among mIF, the AI-based pipeline, and conventional methods, all using CD163 as a marker (ρ = 0.85–0.94) (Fig. 3c, left upper panel). DNA methylation-based deconvolution also showed strong correlation with these methods (ρ = 0.64–0.80), indicating substantial agreement between epigenetic and protein-level quantification of macrophages in pGBs. In contrast, RNA sequencing-based deconvolution exhibited weaker correlations with other methods (ρ = 0.49–0.66). In rGBs, correlation strengths between all methods were generally lower compared to pGBs, with RNA sequencing-based deconvolution producing estimates of weakest correlation (ρ = 0.24–0.77) (Fig. 3c, bottom left panel).

For B cells, marked differences were observed between image-based and in silico-methods. In pGBs, image-based approaches showed strong to very strong concordance (ρ = 0.79–0.93) (Fig. 3c, top middle panel). In contrast, RNA sequencing-based deconvolution showed only weak to moderate correlations with conventional, AI-based pipeline, and HALO-BF methods (ρ = 0.28, 0.48, 0.50, respectively). DNA methylation-based deconvolution exhibited negative correlations with all other methods for B cell quantification in pGBs (ρ = − 0.50 to − 0.06), indicating poor concordance. Although correlations involving DNA methylation-based estimates improved slightly in rGBs, the coefficients remained low (ρ = 0.10–0.35) (Fig. 3c, bottom middle panel). RNA sequencing-based deconvolution also showed weak to moderate concordance with image-based methods in rGBs (ρ = 0.34–0.58). The highest concordance for B cell quantification in rGB samples was observed between HALO-BF and AI-based image analysis (ρ = 0.70).

Similar to the findings for B cells, DNA methylation-based deconvolution estimates for T cell proportions in pGBs showed negative correlations with other methods (ρ = − 0.14–0.04; Fig. 3c, top right panel). RNA sequencing-based deconvolution, mIF, AI-based pipeline, and conventional approaches exhibited weak to moderate correlations for T cell quantification in pGBs (ρ = 0.08–0.68). In rGBs, DNA methylation-based estimates again demonstrated the weakest concordance with other methods (ρ = 0.04–0.27) (Fig. 3c, bottom right panel). The remaining methods showed weak to moderate agreement in T cell quantification, with correlation coefficients ranging from 0.22 to 0.55.

Cross-method concordance was highest for macrophages/monocytes across both tumor states, whereas lymphoid populations exhibited greater variability between imaging- and molecular-based quantification approaches.

### In silico-deconvolution reveals stable non-immune cellular composition and neuroimmune remodeling in rGB

Deconvolution scores for immune and neoplastic cells (Supplementary Fig. 2A-B) were compared between pGB and rGB samples using RNA seq-based deconvolution. Given the inter-method variability in absolute immune cell quantification observed in Fig. 3, subsequent analyses focus on relative differences between pGB and rGB within the same deconvolution framework rather than absolute cell proportions across methods. Using this approach, no significant differences were observed in the abundance of most cell types between the two tumor states, with the exception of macrophages, which were more prevalent in rGB (Supplementary Fig. 2A).

To complement the transcriptomic analysis, DNA methylation-based deconvolution was performed, with cell populations grouped into tissue-resident cells, cancer cells, and immune cells (Supplementary Fig. 2C–D). Consistent with the RNA-based analysis, no statistically significant differences were observed between pGB and rGB across these categories, suggesting a broadly stable cellular composition at the epigenetic level.

To further explore the molecular differences between tumor stages, differential gene expression and Gene Ontology (GO) term enrichment analysis were performed. Overall, the RNA data did not cluster according to tissue type, gender or experimental run (Supplementary Fig. 2E). In pGBs, upregulated genes were associated with cellular proliferation (e.g. *MXD3, MST1*), DNA repair (*TONSL*), and angiogenesis (*NOSTRIN, GJC1*) (Supplementary Fig. 2F). In contrast, rGB samples showed an upregulation of genes related to neurotransmission and synaptic activity (e.g. *GABRA1, GABRG2, PRNP*), as well as immune signaling (*TESPA1*) (Supplementary Fig. 2F). GO term enrichment analysis supported these findings, revealing increased activation of neuronal-related pathways in rGB (Supplementary Fig. 2F).

### Stratification by immune cell infiltration reveals divergent pathway activation in pGB and rGB

**Fig. 4:**
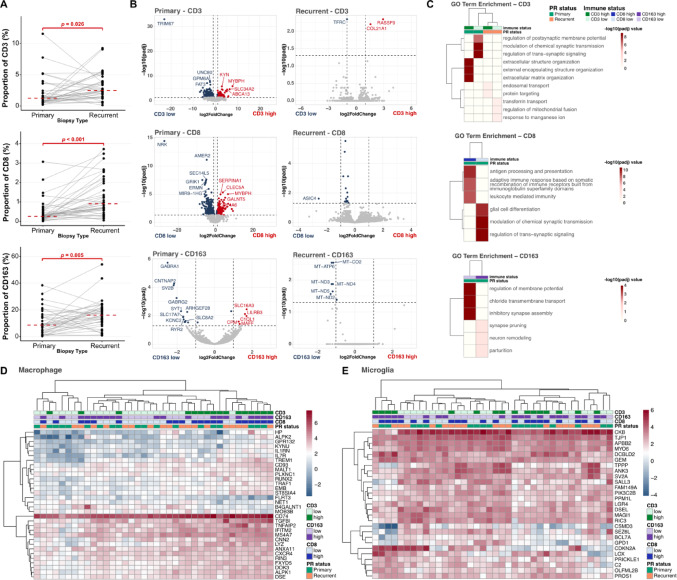
Integrated characterization of immune cell dynamics and transcriptional programs in pGB and rGB. **a** Quantification of immune cell populations by mIF. Dot plots depict the proportion of CD3^+^ T cells, CD8^+^ T cells, and CD163^+^ macrophages in pGB and rGB samples. Statistical significance was assessed using the Wilcoxon signed-rank test. **b** Differential gene expression analysis from RNA sequencing data. Volcano plots display genes differentially expressed between samples with high and low levels (stratified by median) for CD3, CD8, and CD163 immune cell markers in pGB and rGB. **c** Gene Ontology (GO) term enrichment analysis of differentially expressed genes in high vs. low immune cell populations. Bar plots show the top enriched biological processes associated with CD3^+^ T cells, CD8^+^ T cells and CD163^+^ macrophages. Significance is represented as –log10(padj). **d** Heatmap showing the expression of macrophage-related genes across pGB and rGb, stratified by CD3^+^, CD8^+^, and CD163^+^ cell abundance. **e** Heatmap displaying the expression of microglia signature genes in pGB and rGB, stratified similarly by CD3^+^, CD8^+^, and CD163^+^ cell abundance. PR status: primary vs. recurrent. Statistical significance indicated by *p* values as shown

Using multiplex imaging as the reference for cell type estimation, matched pGB-rGB pairs showed significantly higher infiltration of CD3^+^ and CD8^+^ T cells (*p* = 0.026 and *p* < 0.001, respectively), as well as CD163^+^ macrophages (*p* < 0.005) in rGB (Fig. 4a). To further explore these observations, we compared gene expression and GO enrichment in samples stratified by high vs. low infiltration levels of each immune marker.

In pGB, CD3-high tumors upregulated cellular transport, metabolic regulation, and extracellular matrix (ECM) remodeling genes (e.g. *MYBPH*, *KYNU, TGFBI*) (Fig. 4b, top left), whereas CD3-low tumors showed increased neuronal signaling and cell adhesion-related genes (e.g. *TRIM67*, *UNC80*, *FAT3*). In rGB, CD3-high tumors upregulated genes associated with ECM remodeling and epithelial organization (*RASSF9* and *COL21A1*), while CD3-low tumors showed increased *TFRC* expression, a gene involved in iron uptake and cellular metabolism (Fig. 4b, top right). GO enrichment in both tumor types highlighted cellular homeostasis and ECM-related processes in CD3-high samples (Fig. 4c, top panel).

CD8-high pGB samples showed increased immune activation and structural remodeling genes (e.g. *SERPINA1*, *CLEC5A*) and enrichment for antigen presentation and adaptive immunity, whereas CD8-low samples upregulated neuronal signaling genes (*GRIK1*, *AMER2*) involved in glial differentiation and synaptic signaling (Fig. 4b, c, middle left). In rGB, CD8-related transcriptional differences were minimal, with only *ASIC4* differing between groups and no significant GO terms (Fig. 4b, c, middle right).

CD163-high pGB tumors upregulated immune response and inflammation-associated genes (*SLC16A3*, *LILRB3*, *CPM*) (Fig. 4b, bottom left), while CD163-low tumors showed regulation GABAergic (*GABRA1*, *GABRG2*), synaptic (*SV2B*, *SYT1*), and neuronal development genes (*CNTNAP2*) (Fig. 4b, bottom right). GO term reflected membrane potential regulation with synapse assembly in CD163-high samples, and neuron remodeling and synapse pruning in CD163-low samples (Fig. 4c, bottom panel). rGB showed no significant CD163-high changes and only mitochondrial gene upregulation in CD163-low samples (Fig. 4b, c, bottom right).

### Macrophage-related gene expression in pGB and rGB samples

Given the differences in macrophage abundance between pGB and rGB samples, we assessed expression profiles of bone marrow-derived macrophages (BMDMs) and brain-resident microglia-associated signatures (Bowman et al. 2016). Heatmaps of these signatures (Fig. 4d, e) showed that BMDM-associated genes clustered separately by immune infiltration, where high CD3, CD8, CD163-high samples showed upregulated adhesion, migration and signaling genes (e.g. *CD93*, *PLXNC1*, *TREM1*, *MALT1*) (Fig. 4d), whereas low infiltration samples exhibited reduced expression of macrophage polarization genes (e.g. *HP*, *GPR132*, *KYNU*).

Microglia-associated genes were broadly expressed across all tumors, with modest stratification by immune infiltration. In the majority of high CD3, CD8 and CD163 samples, neuronal and metabolic regulation genes (e.g. *CSMD3*, *SEZ6L*, *BCL7A*, *GPD1*) were relatively downregulated, suggesting predominant transcriptional patterns while still capturing heterogeneity within microglial populations.

Overall, GB samples enriched for T-cell and macrophage markers exhibit distinct transcriptional programs involving immune signaling, cellular adhesion, and neuronal-immune interactions.

### Associations of immune markers with disease course

Samples were stratified by sample type, treatment regimen (standard chemo- and radiotherapy vs. non-standard), combined vs. non-combined chemo-radiotherapy, and best therapy response (progressive vs. stable disease) to compare the infiltration of CD3^+^ and CD8^+^ T cells, and CD163^+^ macrophages (Supplementary Fig. 3A-B). Interestingly, no significant differences in immune cell infiltration were observed across any of these clinical subgroups (Supplementary Fig. 3A-B). In CD163 + macrophage levels in rGB however showed a non-significant trend toward lower values in stable disease condition (*p* = 0.058), underscoring inter-patient variability (Supplementary Fig. 3B).

### Longitudinal changes in immune cell populations and DNA methylation profiles in pGB vs. rGB

To assess longitudinal changes associated with GB recurrence, we analyzed both immune cell composition and DNA methylation profiles in matched pGB and rGB samples. Quantification of CD3 + and CD8 + T cells, and CD163 + macrophages revealed a general increase in immune cell infiltration in rGB compared with pGB (Supplementary Fig. 4A).

This increase was observed in majority of samples, with CD8^+^ T cells showing the most consistent rise (81%), followed by CD163^+^ macrophages (69%) and CD3^+^ T cells (67%), while decreases or stable levels were detected in a minority of cases (Supplementary Fig. 4A).

In parallel, matched pGB and rGB samples were subjected to longitudinal DNA methylation profiling, using the Heidelberg Brain Tumor Classifier v11.4/v12.5 (Capper et al. 2018). Transitions between DNA methylation subclasses, including “mesenchymal”, “RTK I”, and “RTK II”, were observed, as well as transitions to or from unclassifiable profiles (Supplementary Fig. 4B). Alterations in the characteristic +7/− 10 chromosomal signature, indicative of GB, were also detected with some recurrent tumors losingof one or both chromosomal changes, while others acquired the hallmark aberration upon recurrence (Supplementary Fig. 4C).

In contrast, *EGFR* amplification status, as inferred from methylation data, remained largely stable across matched samples (Supplementary Fig. 4D). The MGMT promoter methylation status varied between pGB and rGB in a subset of cases or was undeterminable in either the primary or recurrent tumor (Supplementary Fig. 4E).

## Discussion

Despite recent advances in immunotherapy, GB remains the most aggressive form of brain cancer. The current standard treatment consists of maximal surgical resection followed by radiotherapy and chemotherapy. However, disease recurrence is common, with approximately 75.5% of patients relapsing within 8–12 months, and a median post-recurrence survival of 7–12 months (Ostrom et al. 2023; Yoo et al. 2021; Kalita et al. 2023; Vaz-Salgado et al. 2023). Although diagnostic technologies have advanced, individual methodologies remain limited in capturing the full complexity of the rGB TME. To address this, we performed an integrated in situ- and in silico-comparison of paired pGB and recurrent rGB tumors.

### Method comparison

Immune cell profiling showed considerable variation both within and across platforms, emphasizing the need for careful method selection. Among the analyzed cell types, only B cells, T cells, and monocytes/macrophages were comparable across techniques due to differences in marker coverage.

Image-based methods showed concordance for B cells and monocytes/macrophages in pGBs, highlighting their robustness, while DNA methylation-based deconvolution was less consistent. This discrepancy might be likely due to the older 450 K reference signatures, whereas our data was generated using the higher-resolution 850 K array, thus potentially leading to mismatched CpG coverage and reduced accuracy (Chakravarthy et al. 2018). Moreover, the reference dataset was based on CpG sites from CD19^+^ rather than CD20^+^ B cells, which likely contributed to discrepancies in cell type definition. Because CD19 is expressed earlier in B-cell maturation and global methylation patterns change substantially during this process, CD19-derived signatures may capture only early-stage profiles and thus fail to represent the full B-cell spectrum. These markers can distinguish B from non-B cells but do not serve as true pan-B references (Kulis et al. 2015; Forsthuber et al. 2018; Otero and Rickert 2003). Similarly, the limited concordance among in silico methods, particularly for macrophages, may reflect the absence of well-defined, cell-type specific marker signatures in bulk RNA-sequencing datasets (Hegde et al. 2003).

Furthermore, discrepancies between in situ- and in silico-approaches likely reflect the different biological layers they assess: proteomic methods measure total protein abundance, whereas RNA-based approaches capture only transient gene expression and do not reliably predict protein levels (Lee 2016). This discordance is common across cancers, including GB, where transcriptomic and proteomic correlations are often weak (Tang et al. 2018; Lemee et al. 2018), largely due to the complexity of translational regulation (Maier et al. 2009). High inter- and intra-tumoral heterogeneity in GB further amplifies variability in immune cell quantification (Clavreul et al. 2015; Lemee et al. 2015).

### Comparison of cell types identified between pGB and rGB

Across all methodologies, monocyte/macrophage lineage cells were most reliably quantified, highlighting their prominent role within the GB TME (Read et al. 2024; Ho et al. 2024). Their abundance, distinct morphology, and strong marker expression (e.g. CD163) led to greater consistency across platforms compared to T cells (Maddison et al. 2021; Watson et al. 2024). In image-based approaches, AI-driven segmentation pipeline performed best with clearly defined cellular boundaries and consistent antigen expression—criteria more reliably met by macrophages. However, in rGB, the weaker correlation for Iba1^+^ cells between conventional and AI-based readouts may reflect a shift toward highly activated, morphologically complex microglia/macrophages in treatment-altered and perinecrotic regions, which are more difficult to segment robustly and thus accentuate method-specific biases (Bowman et al. 2016; Darmanis et al. 2017). In contrast, T cells, which infiltrate densely and heterogeneously, require more advanced algorithms for accurate quantification (Baharun et al. 2024).

RGB exhibits pronounced histopathological remodeling, including fibrosis, expansion of reactive and resorptive zones, reduced necrosis, and greater preservation of normal brain parenchyma. These structural changes are paralleled by transcriptional upregulation of genes such as *RASSF9* and *COL21A1* in CD3-high tumors, reflecting cytoskeletal reorganization and extracellular matrix remodeling as key features of the post-treatment TME. These structural and molecular changes complicate immune profiling: dense fibrosis impedes antibody penetration and reduces staining fidelity (Bussolati and Leonardo 2008; Stack et al. 2014; Giesen et al. 2014), while whole-slide analyses may be confounded by non-neoplastic tissue. In contrast, AI-based segmentation that excludes fibrotic and normal compartments offer tumor-restricted, biologically meaningful quantification, thereby improving analytical precision and inter-method comparability.

The reduced robustness of immune profiling in rGB is also driven by extensive molecular and epigenetic reprogramming. Longitudinal DNA methylation profiling revealed increased unassignable methylation subclasses and loss of canonical chromosomal signatures, indicating elevated heterogeneity and phenotypic plasticity (Drexler et al. 2024). Although neoplastic cell proportions remained stable between pGB and rGB, their phenotypes shifted substantially, and immune remodeling likely reduces the effectiveness of conventional antibody-based detection, limiting reproducibility, resolution, and interpretability in this complex and evolving disease state (Loussouarn et al. 2023).

### TME reprogramming

The dynamic TME reconfiguration observed in our longitudinal cohort aligns with Varn et al. 2022, showing that glioma evolution is driven by the interplay between tumor-intrinsic genetic alterations and extrinsic immune-mediated pressures. High-resolution mIF revealed concurrent enrichment of CD163^+^ myeloid cells and CD8^+^ cytotoxic T cells at recurrence, a pattern largely missed by conventional IHC, reflecting a spatially heterogenous and potentially checkpoint-restrained immune landscape.

Bulk RNA sequencing supported these observations: recurrent tumors upregulated transcripts linked to synaptic signaling, membrane potential regulation, and vesicle-mediated transport, suggesting neural reprogramming that may facilitate immune evasion and cellular plasticity.

## Limitations

Comparative analyses of the GB TME are often limited by technical variability, including differences in antibody clones, dilutions and staining protocols, which introduce noise and hinder cross-study comparability. To address these challenges, we used a monocentric cohort of matched primary and recurrent GB samples, ensuring consistent tissue processing and immunostaining, thereby enhancing robustness and reducing pre-analytical variability.

Nevertheless, several limitations remain. Marker definition differed across methodologies, e.g. image-based quantification broadly identified CD4^+^ T cells, whereas methylation-based deconvolution quantified CD4^+^ effector T cells. Similar discrepancies were observed for myeloid populations (e.g. CD163 and Iba1 vs. CD14), reflecting differences in resolution, marker specificity, and cell-type definitions across platforms. These challenges underscore the value of multimodal approaches, which enable cross-validation, and more precise cell-type annotation, particularly when combined with high-plex immunofluorescence.

Integrating RNA- and DNA methylation- based deconvolution also depends on accurate reference matrices. GBMdeconvoluteR incorporates primary and recurrent tumor data, yet the evolving cellular landscape may not be fully captured. Additionally, RNA- and methylation assays sampled similar but not identical tumor regions, and spatial heterogeneity of GB likely contributed to some discrepancies. These limitations highlight the need for harmonized protocols and integrative analytical frameworks to improve resolution, reproducibility, and biological interpretability in TME profiling.

## Conclusion

Our cross-methodological analysis of the GB TME highlights persistent challenges in achieving robust immune profiling, particularly in disease recurrence. While monocyte/macrophage-lineage cells were consistently detected, substantial discrepancies in other populations, especially T cells, reflect the limitations of standardized profiling approaches. These challenges are further compounded by the pronounced histological and immunological heterogeneity of rGB.

To improve immune characterization, we advocate for biopsy-specific, cell type-adapted frameworks incorporating AI-driven tumor segmentation for enhanced spatial resolution and analytical accuracy. For tumors with ambiguous molecular features or complex immune architecture, multi-modal strategies integrating spatial proteomics, single-cell analysis, and computational deconvolution provide a more comprehensive and resilient approach. Ultimately, aligning immune profiling methodologies with the dynamic GB microenvironment will be essential for developing more precise and effective immunotherapies.

## Supplementary Information

Below is the link to the electronic supplementary material.

Supplementary Material 1

Supplementary Material 2

Supplementary Material 3

Supplementary Material 4

## Data Availability

All count data generated in this study are available in the supplementary data section (Supplementary Excel File 1, 2 and 3). All other data, including raw data, can be shared upon request.

## Abbreviations

AC: Astrocyte-like
BMDM: Bone marrow-derived macrophage
BF: Brightfield
CCNU: Lomustine
DC: Dendritic cell
EGFR: Epidermal growth factor receptor
FFPE: Formalin-fixed paraffin-embedded
GO: Gene ontology
IHC: Immunohistochemistry
mIF: Multiplex immunofluorescence
MES: Mesenchymal
MGMT: O6-methylguanine-DNA methyltransferase
NPC: Neuronal progenitor-like
OPC: Oligodendrocyte progenitor-like
padj: Adjusted *p*-value
pGB: Primary glioblastoma, IDH wildtype, CNS WHO grade 4
rGB: Recurrent glioblastoma, IDH wildtype, CNS WHO grade 4
Rthx: Radiation therapy
TMZ: Temozolomide
TME: Tumor microenvironment
TSA: Tyramide signal amplification
UCT: University Cancer Center
Nivo: Nivolumab
TPM: Transcript per million
