# Supplementary material for: Comparative cross-methodological analysis of the IDH-wildtype glioblastoma tumor microenvironment
Source: J Cancer Res Clin Oncol. 2026 Feb 28;152(2):53. doi: 10.1007/s00432-026-06428-6 (PMC12950149; doi:10.1007/s00432-026-06428-6)
Supplement: Supplementary file 4 — Supplementary Material 4 [file 432_2026_6428_MOESM4_ESM.docx]

**Supplementary Figures**

**
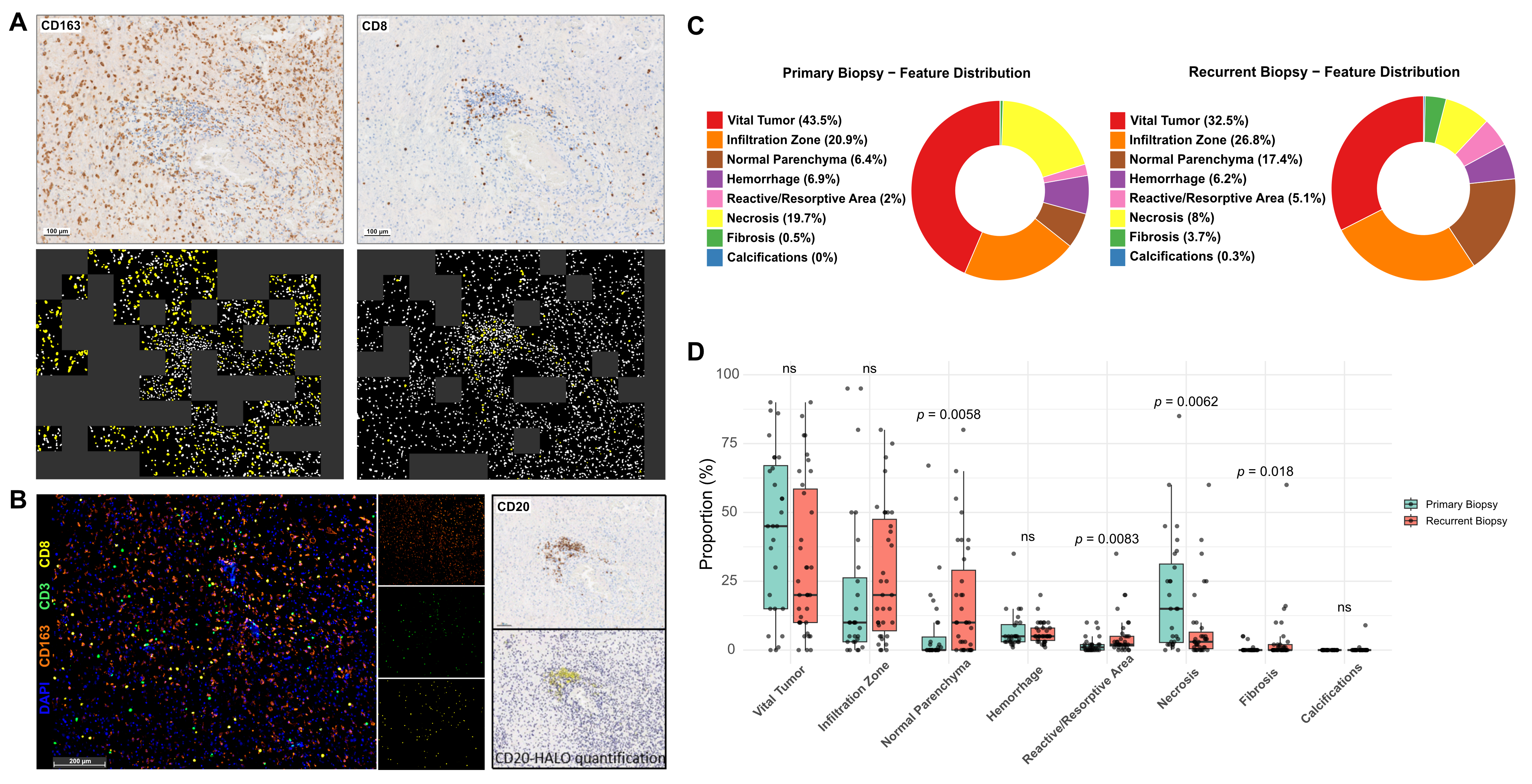
**

**Supplementary Fig. 1. Comparative tissue and immune cell analysis in GB using IHC and mIF approaches.**

**A** Representative IHC images showing CD163^+^ macrophages and CD8^+^ T cells (top), with the corresponding quantification output from the AI-driven pipeline (bottom) for the same sample. B A mIF image illustrating the spatial distribution of immune cell subsets, including CD3^+^ T cells, CD8^+^ cytotoxic T cells, and CD163^+^ macrophages, is shown on the left. CD20⁺ B cell counts were obtained from IHC-stained sections and quantified using the HALO® image analysis platform, with the corresponding output shown on the right. **C** Distribution of histopathological features in pGB and rGb samples based on H&E-stained sections. Light microscopy was used to estimate the proportions of vital tumor area, infiltration zones, normal parenchyma, hemorrhage, reactive/resorptive areas, necrosis, fibrosis, and calcification. **D** Comparative analysis of histopathological features between pGB and rGB samples.


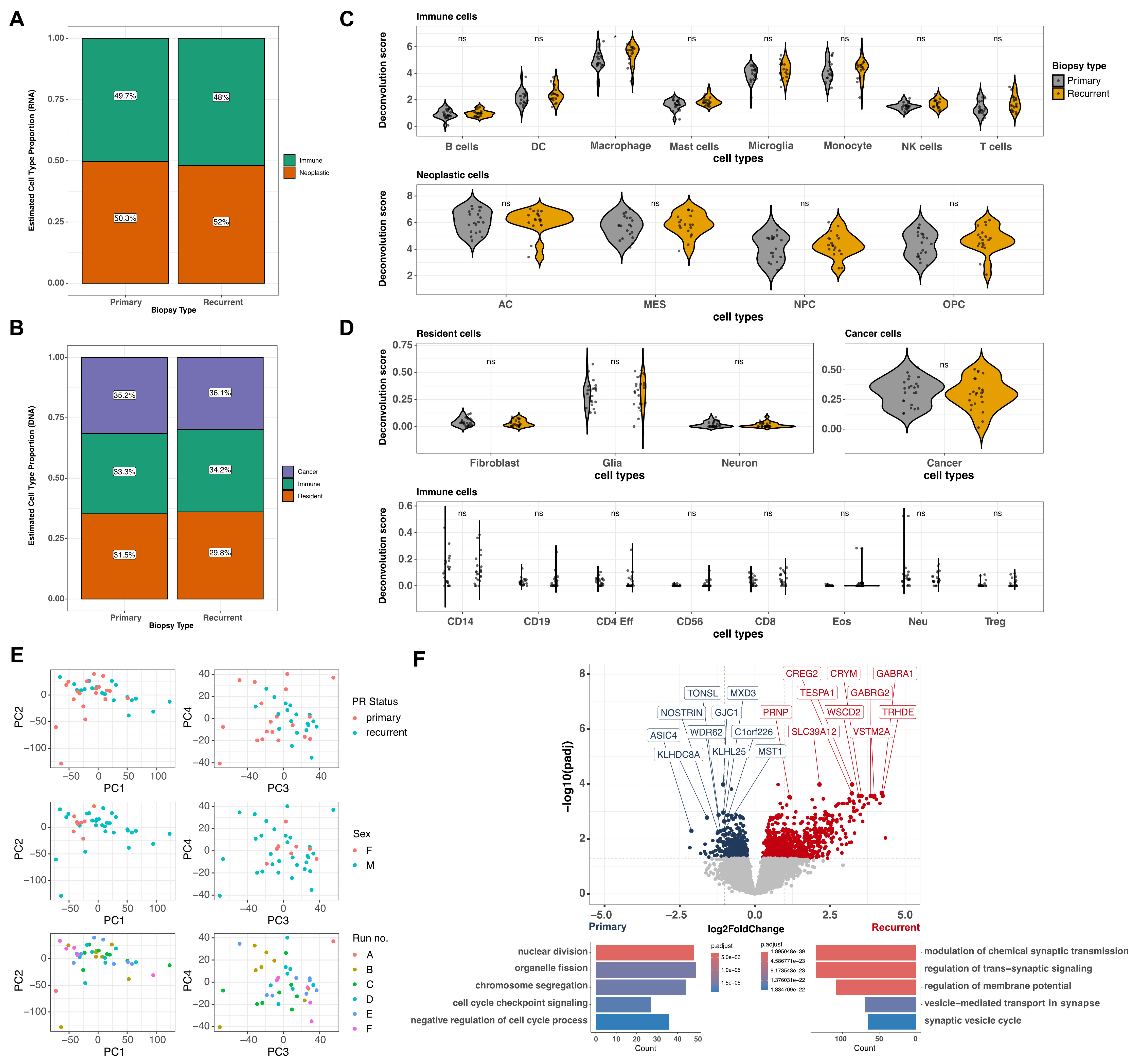


**Supplementary Fig. 2. Deconvolution of immune and neoplastic cell types and differential gene expression analysis in pGB and rGB samples. A** Deconvolution of bulk RNA sequencing data was performed using **‘GBMdeconvoluteR’** to estimate cell type proportions. Bar plot represents estimated cell type proportions of immune cells and neoplastic cell types in pGB and rGB samples. **B** DNA methylation data was subjected to deconvolution to assess cellular composition. Estimated proportions for tissue-resident brain cells, neoplastic cells, and immune cell populations are shown in the stacked bar graph. **C** Bulk RNA deconvolution scores for immune cells (top) and neoplastic cell types (bottom) were compared between pGB (grey) and rGB (dark yellow) samples. Statistically significant differences (p < 0.05) are indicated with an asterisk (*); non-significant comparisons are labeled as "ns". **D** Violin plots display estimated proportions for tissue-resident brain cells (upper left), neoplastic cells (upper right), and immune cell populations (bottom). Group comparisons between pGB and rGB follow the same color scheme and significance notation as in panel C. **E** Principal component analysis of bulk RNA sequencing transcriptional profiles of pGB and rGB samples. Different PCs are displayed to show the distribution of primary/recurrent status, sex and run numbers. **F** Volcano plot (top) showing differentially expressed genes between pGB and rGB samples. The top 10 most significantly upregulated or downregulated genes (log_2_ fold change > 2, padj < 0.05) are labeled. GO term enrichment analysis (bottom) was performed on significantly differentially expressed genes (padj < 0.05; log_2_ fold change > 0.5 or < -0.5). Enriched biological processes are shown, sorted by descending padj values, as indicated by the legend.


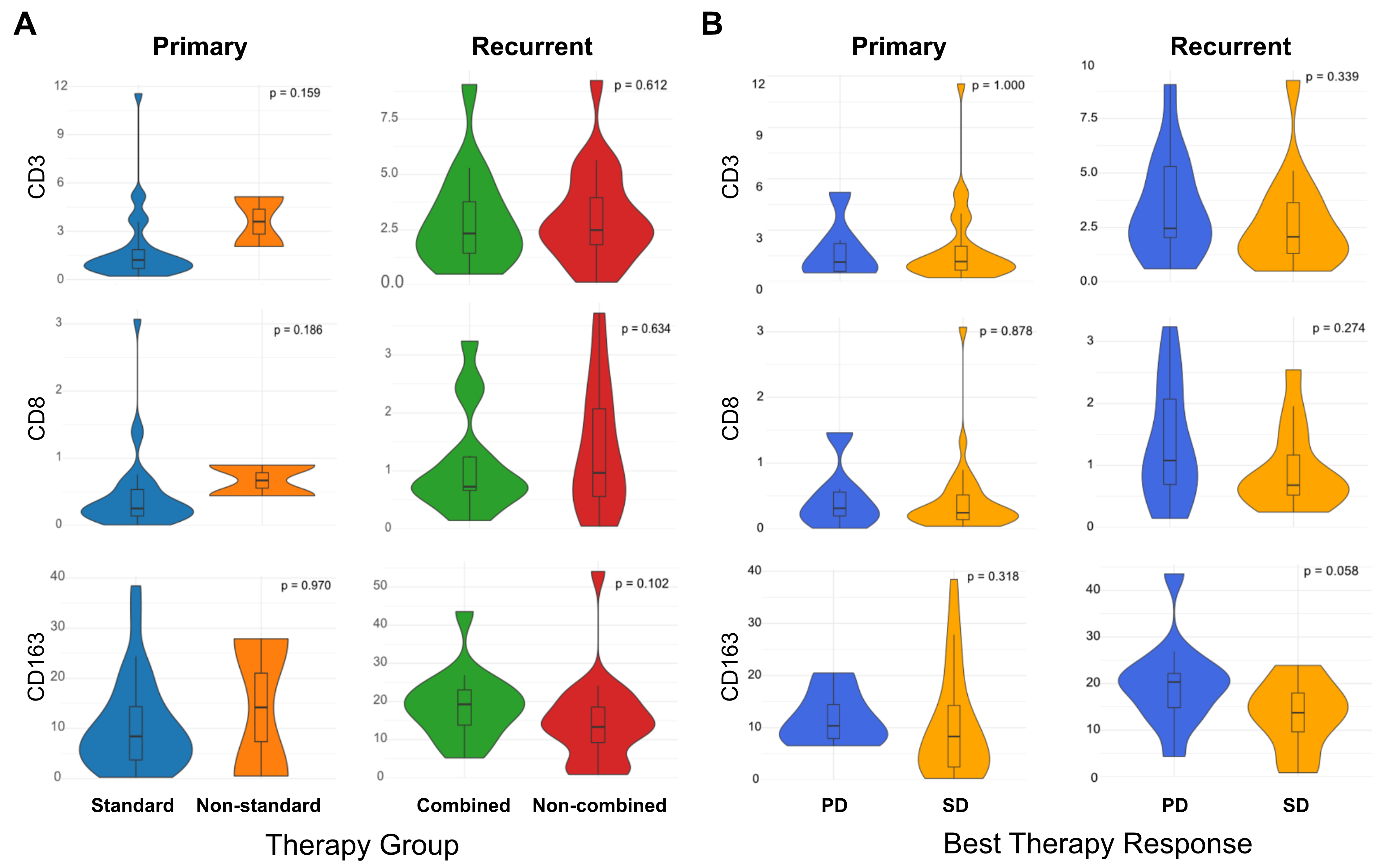


**Supplementary Fig. 3. Distribution of immune markers across treatment groups and response categories. A–B** Quantification of CD3^+^, CD8^+^, and CD163^+^ immune cell populations using mIF, stratified by (A) treatment group and (B) therapy response categories. Violin plots illustrate the distribution of expression levels for each marker. Statistical comparisons between groups were conducted using the Wilcoxon rank-sum test, with exact *p*-values displayed above each comparison.


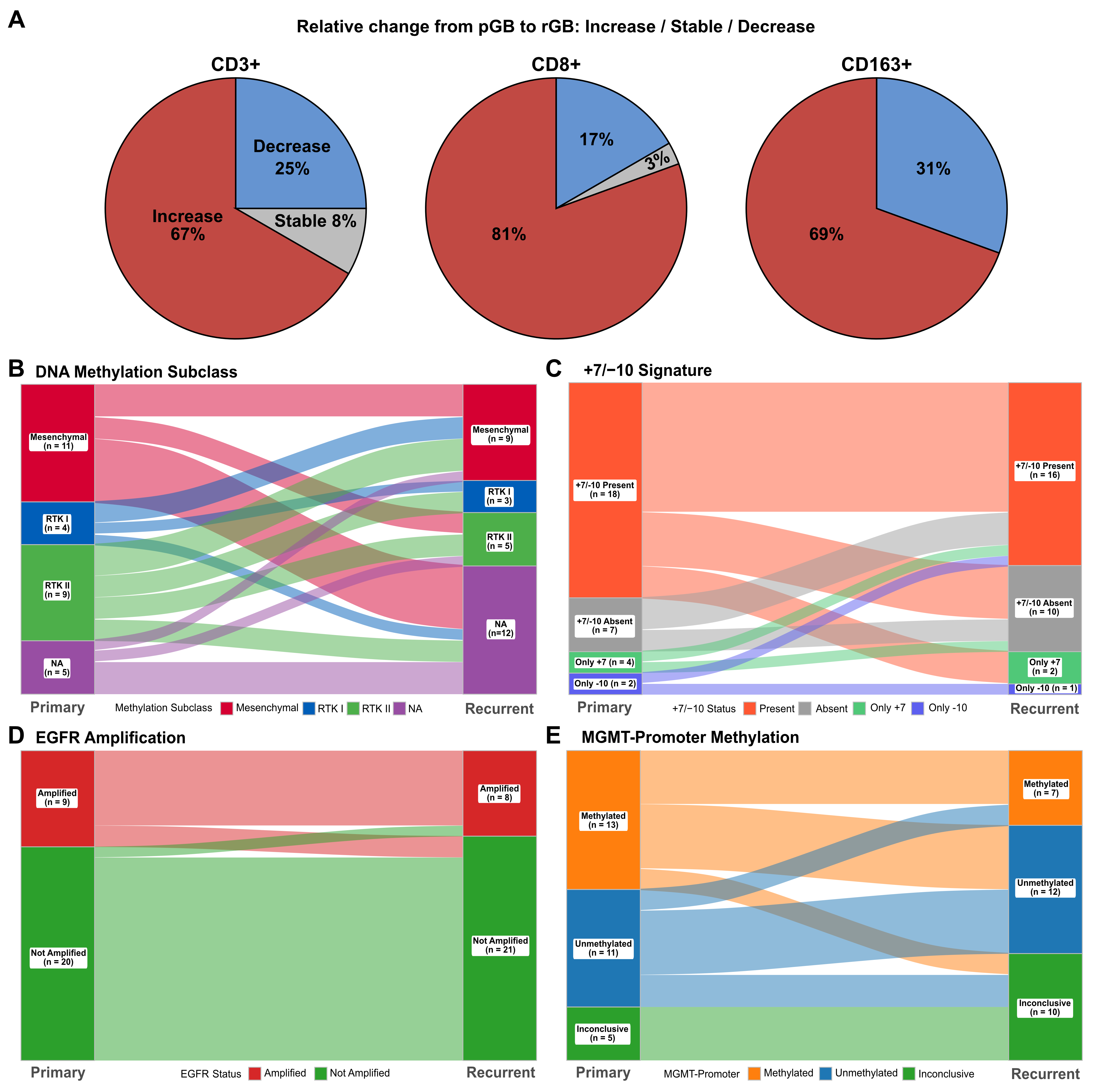


**Supplementary Fig. 4. Comparative DNA methylation profiling in pGB vs. rGB A** Pie charts illustrating the percentage of samples with increased (red), decreased (blue), or stable (gray) immune cell levels (CD3, CD8 and CD163) from pGB to rGB (10% relative difference, respectively). **B**  Sankey diagrams illustrate shifts from pGB to rGB in DNA methylation subclass allocation (mesenchymal, RTK I, RTK II, not assigned [NA]), **C** +7/-10 chromosomal signature status (+7/-10 present, +7/-10 absent, only +7, only -10), **D** *EGFR* amplification status (amplified, not amplified), and **E** MGMT promoter methylation status (methylated, unmethylated, inconclusive). The width of each flow is proportional to the number of samples transitioning between categories, providing a visual representation of molecular changes between pGB and rGB samples.
